# Predatory Capacity and Reproduction of *Cyrtorhinus lividipennis* (Hemiptera: Miridae) Adults Exposed to Low-Temperature Storage and Fitness of the F1 Generation

**DOI:** 10.3390/insects14030226

**Published:** 2023-02-24

**Authors:** Yuqi Zhong, Xiaolan Liao, Maolin Hou

**Affiliations:** 1 State Key Laboratory for Biology of Plant Diseases and Insect Pests, Institute of Plant Protection, Chinese Academy of Agricultural Sciences, Beijing 100093, China; 2 College of Plant Protection, Hunan Agricultural University, Changsha 410028, China

**Keywords:** *Cyrtorhinus lividipennis*, predation, reproduction, fitness, low temperature storage, biological control

## Abstract

**Simple Summary:**

*Cyrtorhinus lividipennis* Reuter (Hemiptera: Miridae) is an important predator of planthoppers and leafhoppers in rice fields. Augmentative biological control has been practiced successfully in many agroecosystems. However, one of the primary obstacles to augmentative biological control is obtaining natural enemies in sufficient numbers and quality when required for release. The development of the low-temperature storage (LTS) technique has been pivotal in ensuring the flexibility and efficiency of the mass production of biological control agents. Here, we measured the effects of LTS on the predatory capacity and reproduction of *C. lividipennis* adults and the fitness of the F1 generation. The results are expected to improve the successful utilization of the predator in an IPM program.

**Abstract:**

Low-temperature storage (LTS) is a way to adjust natural enemy development to meet field release needs and to protect natural enemies from the odds of long-distance transportation. The mirid bug *Cyrtorhinus lividipennis* Reuter (Hemiptera: Miridae) is an important predator of planthoppers and leafhoppers in rice fields. In this study, the LTS effects were measured on the predatory capacity and reproduction of the mirid adults (provided with 20% honey solution and stored at 13 °C for 12 days), and the fitness of the F1 generation of these adults. Higher predation of the eggs of the brown planthopper *Nilaparvata lugens* (Stål) (Hemiptera: Delphacidae) was observed in the post-storage females than in the control females. The functional responses of *C. lividipennis* adults, either exposed to LTS or not, to planthopper eggs fitted well with Holling type II functional responses. Longevity was not affected by LTS, whereas the number of offspring nymphs was 55.6% lower in the post-storage females than in the control females. The fitness of the offspring generation was not affected by the LTS of parental adults. The findings are discussed with their relevance to biological control.

## 1. Introduction

The mirid bug, *Cyrtorhinus lividipennis* Reuter (Hemiptera: Miridae: Cimicomorpha: Miridae: Orthotylinae), is an important predator widely distributed from tropical to warm temperate rice production regions, preying on the eggs and young nymphs of planthoppers and leafhoppers [1], such as the migratory brown planthopper *Nilaparvata lugens* (Stål) and white-backed planthopper *Sogatella furcifera* (Horvath), and the green leafhopper *Nephotettix virescens* (Distant), that are destructive rice insect pests. The mirid bug cannot overwinter locally in the subtropical and temperate regions, and local occurrence depends on annual migration from the tropical regions with the planthoppers [2]. Due to its high predatory capacity, *C. lividipennis* appears as a promising biocontrol agent against these hopper pests [3,4,5,6]. The improvement in the mass-rearing techniques of *C. lividipennis* has made it possible to include the predator’s augmentative release in any rice integrated pest management (IPM) program [7,8].

Biological control is a key component of any IPM package. Augmentative biological control has been practiced successfully in many agroecosystems, which can be realized through inundative release or inoculative release of mass-produced natural enemies [9]. However, one of the primary obstacles to augmentative biological control is the difficulty of obtaining natural enemies in sufficient numbers and quality when they are required for release [10]. The short shelf life of the produced natural enemies makes it impossible to store them under normal conditions, while demands for them usually vary between seasons and due to changes in weather conditions [11]. Further, the cost of maintaining natural enemy colonies is remarkably high when they are not in demand [10]. Moreover, transportation of the natural enemies from insectaries to the sites of release can threaten their biological control service. All these factors point to the development of the low-temperature storage (LTS) technique that is pivotal in ensuring the flexibility and efficiency of mass production of biological control agents [11]. Nevertheless, LTS may impose costs on biological control services for the stored natural enemies or costs on offspring fitness [11].

Our previous investigation has found that supplementary food and low temperatures can significantly extend the lifespan of *C. lividipennis* [6]; the adults stored at 13 ± 1 °C for 12 days with the supplementary nutrient of 20% honey solution could survive at a rate of more than 80%. The temperature of 13 °C is close to the low temperature of early spring, which would support the possibility of early spring release. The migration of *C. lividipennis* is usually later than that of planthoppers [12], so the establishment of a local *C. lividipennis* population may play an essential role in controlling planthoppers. The present study was designed to measure the effects of LTS on the predatory capacity and reproduction of *C. lividipennis* adults and the fitness of the F1 generation. The results are expected to improve the successful utilization of the predator in an IPM program.

## 2. Materials and Methods

### 2.1. Insects and LTS Treatment

A laboratory stock culture of *C. lividipennis* was established from collections in the paddy fields at Guilin Experiment Station for Crop Pests (25°36′00″ N and 110°41′24″ E), Ministry of Agriculture and Rural Affairs, China. The insects were reared on brown planthopper eggs deposited in 10 d old rice seedlings (var. TN1) enclosed in a 100-mesh rearing cage (60 cm × 60 cm × 60 cm). Newly emerged (<24 h) *C. lividipennis* adults were paired in plastic cups (5 cm × 18 cm) with a ball of cotton wool soaked with 20% honey solution and stored at 13 ± 1 °C for 12 days in a climate chamber (0 L:24 D, RH 70 ± 10%). The honey solution in the cotton wool was replaced every two days.

### 2.2. The Predatory Capacity of Post-Storage C. lividipennis Adults

To detect LTS effects on the predatory capacity of *C. lividipennis* adults, the post-storage adults starved for 24 h in the same climate chamber (26 ± 1 °C, 14 L:10 D, RH 70 ± 10%) were individually placed in glass tubes (2 cm× 8 cm) with a moistened cotton wool at the bottom and a sponge plug at the opening. The predators were provided with rice leaf sheaths deposited within 48 h with brown planthopper eggs, which were obtained by confining 1000 gravid brown planthopper females in a 100-mesh cage with 10 d old TN1 rice seedlings cultured in a plate (42 cm× 28 cm). The leaf sheaths were dissected under a stereomicroscope for the number of brown planthopper eggs therein so that there were 20, 30, 40, 60, and 80 eggs per tube for *C. lividipennis* males and 30, 40, 50, 70, and 90 eggs per tube for females. Newly emerged (≤24 h) *C. lividipennis* adults maintained at 26 °C and starved for 24 h were used as the control. The *C. lividipennis* adults were left to predate on brown planthopper eggs in the climate chamber for 24 h, and then the number of eggs predated was checked under a stereomicroscope. The eggs that were shriveled or left with eggshells were considered as being predated. For each treatment combination (prey density × LTS), the observation was repeated 5 times. In this measurement, the predated eggs were not replenished, according to the procedure for the predatory mite, *Neoseiulus cucumeris* (Oudemans) (Acari: Phytoseiidae) [13] and the two-spot ladybird, *Adalia bipunctata* (L.) (Coleoptera: Coccinellidae) [14].

### 2.3. Reproduction of Post-Storage C. lividipennis Adults

To measure LTS effects on *C. lividipennis* reproduction, the post-storage *C. lividipennis* adults were paired in plastic cups (5 cm × 18 cm) with two 10 d old rice seedlings and a pair of brown planthopper female and male adults (48 h old) that served to supply prey eggs. The cups were sealed at the opening with nylon gauze and then transferred to the climate chamber. The cups were checked each day for the survival of *C. lividipennis* and planthopper adults; upon the death of *C. lividipennis* females, the observation was stopped; in the case of death of *C. lividipennis* males or brown planthopper adults, the respective insects were replenished. Every two days, the *C. lividipennis* adults were moved to a new cup until the death of the *C. lividipennis* females. The replaced cups and seedlings were maintained in the climate chamber. Six days after replacement, the cups were checked daily for 10 days for the hatching of *C. lividipennis* nymphs, and the nymphs, if any, were counted and removed from the cups. *C. lividipennis* female longevity and number of nymphs per female were calculated. The control *C. lividipennis* adults were observed in the same way. Fifteen pairs of the post-storage or control *C. lividipennis* adults were initially established for observation.

### 2.4. Fitness of F1 Generation of Post-Storage C. lividipennis Adults

To investigate LTS effects on the fitness of the F1 generation of the post-storage *C. lividipennis* adults, newly hatched *C. lividipennis* nymphs resulting from the post-storage and the control adults were individually reared in plastic cups with two 10 d old rice seedlings and a pair of brown planthopper female and male adults (48 h old) in the same climate chamber (26 ± 1 °C, 14 L:10 D, RH 70 ± 10%). The *C. lividipennis* nymphs were observed daily for survival and molting, and the planthopper adults were replenished upon death. Upon emergence, the offspring of *C. lividipennis* adults were sexed. Sixty *C. lividipennis* nymphs derived from the post-storage or the control females were initially observed. Developmental duration, emergence rate, and sex ratio of the F1 generation *C. lividipennis* were calculated.

### 2.5. Data Analyses

The effects of prey density, LTS, and their interaction on the predation rates of *C. lividipennis* adults on brown planthopper eggs were analyzed using general linear models (GLM). The predation rates were calculated from the number of preys consumed and the number of preys provided. Before GLM, data on the predation rates were arcsine square root transformed for homogeneity consideration. The main effects of prey density on the predation rate of female and male mirid bugs were separated by the Tukey test.

To determine the type of functional response of *C. lividipennis* to brown planthopper eggs, logistic regression (Equation (1)) of the proportion of preys consumed in relation to initial prey density using a logit link function was conducted to fit the obtained data with SAS software 9.4.

(1) $$\frac{N_{e}}{N_{0}}=\mathrm{Prob}\;(Y=1)=\frac{\exp\left(P_{0}+P_{1}N_{0}+P_{2}N_{0}^{2}+P_{3}N_{0}^{3}\right)}{1+\exp\left(P_{0}+P_{1}N_{0}+P_{2}N_{0}^{2}+P_{3}N_{0}^{3}\right)}$$

where *N_e_* is the number of preys consumed and *N*_0_ is the initial prey density. Y is dichotomous, which represents prey being consumed (Y = 1) or prey still alive (Y = 0) at the end of the experiment. *P*_0_, *P*_1_, *P*_2_, and *P*_3_ are the maximum likelihood estimates of the intercept, linear, quadratic, and cubic coefficients, respectively. The type of functional response was determined by the signs of the linear and quadratic coefficients. If the linear coefficient is significantly negative, the predator displays a Type II functional response, which indicates that the proportion of prey consumed declines monotonically with the initial prey density. When the linear term is positive, and the quadratic term is negative, the predator displays a Type III functional response [15]. Given that logistic regression analysis indicated that our data fitted with Type II functional response in each case, further analysis by the disc equation [16,17] was conducted to obtain the values of attack rate (α) and handling time (*T_h_*). A nonlinear least square regression (Equation (2)) procedure was used to estimate the α and *T_h_* of the random predator equation [14] by GraphPad software (GraphPad Prism 8.0.1, San Diego, CA, USA).

(2) $$Ne=\frac{\alpha N_{0}T}{1+\alpha T_{h}N_{0}}$$

where *N_e_* is the number of preys consumed, *N*_0_ is the initial prey density, *T* is the total exposure time (24 h), *α* is the attack rate, and *T_h_* is the handling time. The parameters *α* and *T_h_* were considered significantly different between the post-storage and control *C. lividipennis* adults if 95% confidence intervals were not overlapping. 

Differences in *C. lividipennis* adult longevity and fecundity and offspring nymphal developmental duration were compared using an independent sample t-test between the control and the post-storage group, and differences in offspring emergence rate and sex ratio between the control and the post-storage group were detected using the Marascuillo procedure [18].

## 3. Results

### 3.1. The Predatory Capacity of Post-Storage C. lividipennis Adults

**Figure 1:**
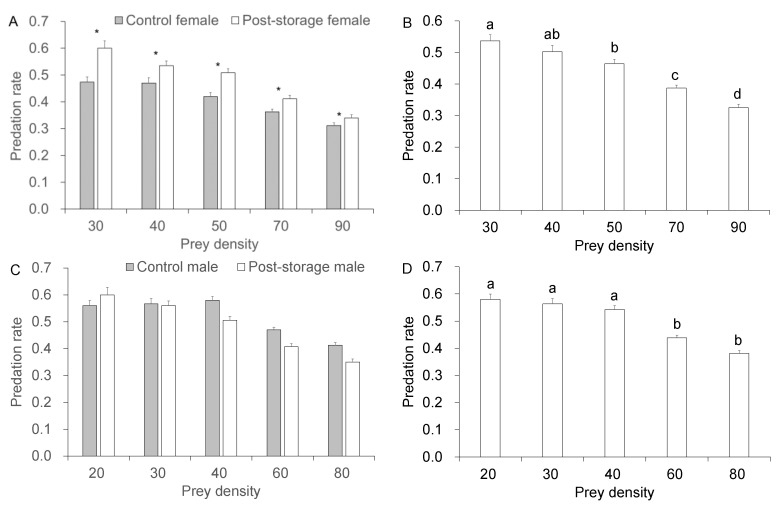
Predation of *Nilaparvata lugens* eggs by post-storage and control *Cyrtorhinus lividipennis* adults. (**A**) *C. lividipennis* female, (**B**) main effect of prey density in females, (**C**) *C. lividipennis* male, and (**D**) main effect of prey density in males. The data represent mean ± SE. * shows a significant difference (independent sample *t*-test, *p* = 0.05). Different letters over the bars indicate a significant difference (Tukey test, *p* = 0.05).

LTS showed a significant influence on the predation rate of *C. lividipennis* female adults on brown planthopper eggs (*p* < 0.001, Table 1) but not on the predation rate of *C. lividipennis* male adults on planthopper eggs (*p* = 0.098, Table 1). Post-storage *C. lividipennis* females exhibited a higher predation rate than the control females (Table 1 and Figure 1A). For both post-storage and control *C. lividipennis* adults, the predation rate decreased with an increase in prey density (Figure 1A,C). Significantly higher predation rates were found at low prey densities (Figure 1B,D). However, there were no significant effects of the interaction between LTS and prey density on the predation rates of both *C. lividipennis* sexes (*p* = 0.061 for females and *p* = 0.302 for males, Table 1). 

**Figure 2:**
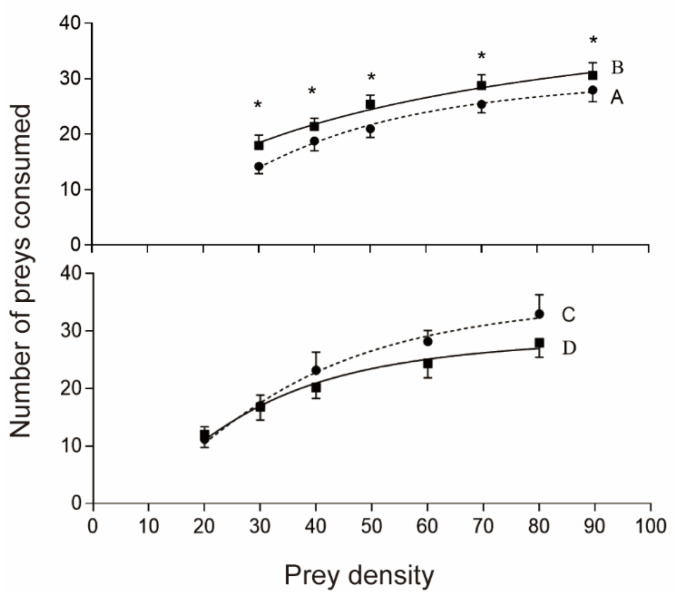
Functional responses of post-storage and control adults of *Cyrtorhinus lividipennis* to *Nilaparvata lugens* eggs. A: control females, B: post-storage females, C: control males, and D: post-storage males. The data (mean ± SE) represent the numbers of preys consumed by the predator in 24 h; the lines are fitted using the Holling type II equation. * shows a significant difference (independent sample *t*-test, *p* = 0.05).

The number of preys consumed by both females and males of the predator whether exposed to LTS or not was drawn to initial prey density, which showed a logistic regression pattern (Figure 2). Coefficients of the linear terms of the logistic regression of the number of preys consumed in relation to initial prey density were significantly negative (*p* < 0.05; Table 2), indicating that the functional responses are of Type II. Estimates of parameters of attack rate (α) and handling time (*T_h_*) by the Holling disc equation are presented in Table 3. Based on the 95% confidence intervals, the attack rate α differed significantly between the post-storage and the control *C. lividipennis* adults of both sexes (Table 3). Both post-storage females and males showed higher attacking rates on brown planthopper eggs than the control insects (Table 3). The handling time was not different between the post-storage and the control females but was more prolonged in the post-storage males than in the control males.

### 3.2. Reproduction of Post-Storage C. lividipennis Adults

**Figure 3:**
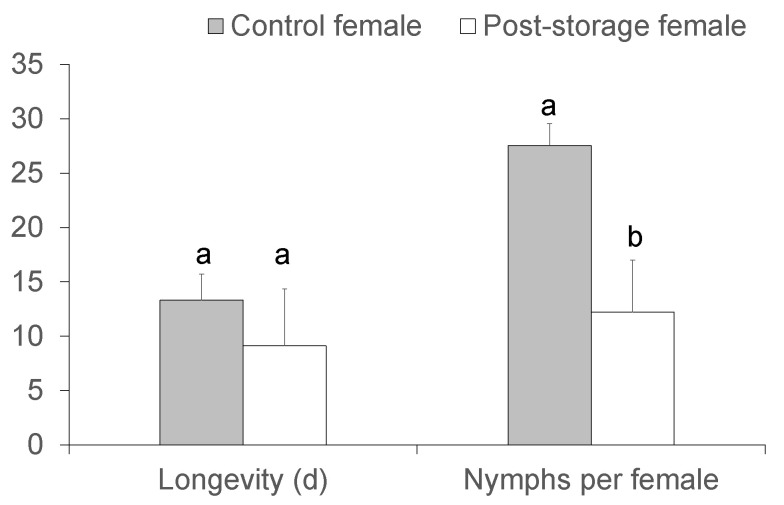
Longevity and nymphs per female of post-storage and control *Cyrtorhinus lividipennis* female adults. The data represent mean ± SE. Different letters over the bars indicate significant differences (independent sample *t*-test, *p* = 0.05).

The longevity of post-storage *C. lividipennis* female adults was not significantly reduced in comparison with that of the control females (*t* = 1.344, df = 28, *p* = 0.190). The number of nymphs per female was significantly reduced by 55.6% in the post-storage compared to the control females (*t* = 2.153, df = 28, *p* = 0.040) (Figure 3). 

### 3.3. Fitness of F1 Generation of Post-Storage C. lividipennis Adults

**Figure 4:**
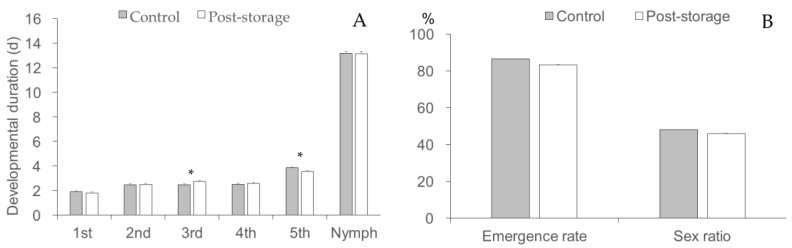
(**A**) Developmental duration; (**B**) emergence, and sex ratio (F1 generation of post-storage and control Cyrtorhinus lividipennis adults). The data represent mean ± SE. * shows a significant difference (independent sample *t*-test, *p* = 0.05).

*Cyrtorhinus lividipennis* offspring fitness was generally not affected by the LTS of parental adults (Figure 4). Nymphal developmental duration (about 13 days) was not different between the control and the post-storage group (*t* = 0.135, df = 100, *p* = 0.894), although there were differences in the durations of the third and fifth instars (*t* ≤ 2.631, df = 100, *p* ≤ 0.010). Offspring emergence rate (83−87%) and sex ratio (46−48% of females) were also not different between the control and the post-storage group (Marascuillo procedure).

## 4. Discussion

Low-temperature storage is essential for augmentative biological control in that it adjusts natural enemy development to meet field release needs and protect predators from the hazards of long-distance transportation [11,19]. Low temperatures usually reduce the metabolism of the exposed natural enemies and thus prolong their survival but may affect the biological control service and reproduction of the exposed natural enemies and the fitness of their offspring [6,10,11]. Although important for augmentative biological control in general, the topic is studied more in parasitoids than in predators [11].

The influence of cold storage on natural enemies is usually assessed for both the exposed parental generation and the F1 generation for its fitness and biocontrol service. For the biocontrol service of the parental generation, the post-storage female adults of *C. lividipennis* in the present study showed a significant increase in predation rate on brown planthopper eggs of 13.3% over the control females. Similar results were reported in the predatory mite *Neoseiulus californicus* (McGregor), where the post-storage female mites predated more than the control females [20], and in the ladybird *Harmonia axyridis* (Pallas), where the individuals that were stored for 150 days at 3 °C consumed significantly more aphids than the unstored ones. It is possible that after being kept for 12 days on carbohydrate food only they must consume a certain amount of energy reserves to maintain their metabolism and/or produce cryoprotectant compounds, such as myoinositol [21]. In contrast with the females, the post-storage male adults experienced no change in predation rate compared to the control males. The differences in attack rate (α) and handling time (*T_h_*) between post-storage and control adults may explain the differences in their predation rate. A high attack rate (α) and short handling time (*T_h_*) are often good indicators of a high predation rate [22]. The higher attack rate in the post-storage females than their control counterparts, coupled with no difference in handling time between them, pinpoints the higher predation rate in the post-storage females than the control females. The higher attack rate but longer handling time in the post-storage males than their control counterparts explain the lack of difference in predation rate between them. 

Cold storage usually entails fitness costs in the exposed parental generation of IPM predators [11,23,24,25]. In this study, LTS did not influence the longevity of the post-storage *C. lividipennis* adults but significantly reduced the number of offspring nymphs per female by 55.6% in the post-storage females compared with the control females. A similar fecundity cost has been observed in most post-storage parasitoids and predators, such as the wasp parasitoid *Habrobracon hebetor* Say [23], two olive fruit fly parasitoids, *Psyttalia humilis* (Silvestri) and *P. ponerophaga* (Silvestri) [26], the fly parasitoids *Exorista japonica* (Townsend) [24] and *E. larvarum* (L.) [19], the predatory mite *N. californicus* [20], and the green lacewing *Chrysopa pallens* Wesmael [25], among the others. By contrast, *H. axyridis* beetles post-storage of 120 days at 6 °C have similar fecundity as the control beetles [27]. Compromised longevity resulting from LTS is also reported in many natural enemies, including *E. larvarum* [19], *H. hebetor* [23], and *E. japonica* [24]. The mechanisms for the fitness costs observed with LTS are complex but are apparently connected with chilling injury, which can arrest ovarian development, reduce the activities of protease, lipase, and trehalase [25], hinder membrane lipid phase transition, and disturb ion homeostasis [28]. This is especially true in arthropods not entering diapause or experiencing cold acclimation before cold storage [28], such as *C. lividipennis*. By contrast, cold storage of pre-wintering *H. axyridis* resulted in no noticeable reduction in fitness [21]. Nutrient consumption during cold storage may also reduce the fitness of the cold-exposed natural enemies. The insects consume much fat when stored at low temperatures for a long time for maintenance, which is at the cost of reproduction and survival [29]. 

Fitness costs associated with LTS can occur cross-generation. Colinet and Boivin [11] have summarized most cases of trans-generational effects of LTS in parasitoids. Our results indicate that LTS of *C. lividipennis* adults imposed no fitness cost on developmental duration, emergence rate, and sex ratio of the offspring nymphs. In the F1 progeny of *Encarsia formosa* (Gahan) and *Eretmocerus corni* (Haldeman) pupae that experienced cold storage, fitness costs are also not observed [30]. The F1 offspring of *H. axyridis* beetles exposed to long-term storage at 6 °C showing a significantly reduced egg hatch rate [25]. The mechanism of trans-generational effects of cold storage is not precisely known, though chilling during cold storage of adults may damage the immature oocytes or change maternal metabolism [31]. 

Since cold storage can impose fitness costs on both the post-storage parental generation and their offspring, it is essential to improve the cold tolerance of insects during storage. Many factors have been indicated to influence the cold tolerance of insects before, during, or even after cold storage [11], including exogenous factors such as diet [10,29], relative humidity [32], temperature [26], acclimation [33,34], and chemicals [25,35], and endogenous factors such as stage [35]. Colinet and Boivin [11] have detailed these factors for insect parasitoids, which broadly apply to predators. It should be noted that cold tolerance is a plastic trait that can vary greatly in an insect in response to changes in environmental factors or between species in response to the same factor. Therefore, it is essential to understand the effects of the exogenous and endogenous factors on cold tolerance case by case to store natural enemies at low temperatures successfully.

LTS can significantly slow down the growth and development of natural enemy insects, reduce their metabolic rate, and extend their shelf life. The present study reveals that LTS increases predatory capacity in *C. lividipennis* but imposes a fertility cost on the post-storage females and does not significantly affect the predatory capacity of males, the life span of the post-storage parental insects, and the fitness of the offspring. These results indicate that the post-storage *C. lividipennis* adults, when released in an inundative program, can achieve comparable or even better biological control service than the individuals not experiencing cold storage. However, due to their reduced fertility, the post-storage mirid bugs may be unsuitable for inoculative purposes. Additional studies are needed to optimize the LTS conditions, such as acclimation, chemicals, and environmental factors, to reduce the associated fertility cost and to understand the mechanisms behind the fertility cost.

## 5. Conclusions

In summary, we observed higher predation of eggs of the brown planthopper *Nilaparvata lugens* in the post-storage *Cyrtorhinus lividipennis* females than in the control females. Longevity was not affected by the low-temperature storage. By contrast, the number of offspring nymphs was reduced by 55.6% in the post-storage *Cyrtorhinus lividipennis* females compared to the control females. The fitness of the *Cyrtorhinus lividipennis* offspring generation was not affected by the low-temperature storage of parental adults. These results indicate that the post-storage *C. lividipennis* adults, when released in an inundative program, can achieve comparable or even better biological control service than the insects not experiencing cold storage. However, due to their reduced fertility, the post-storage mirid bugs may be unsuitable for inoculative purposes.

## Figures and Tables

**Table 1:** Results of general linear models (GLM) analyzing the effects of low temperature storage (LTS) and prey density on the predation rates of *Cyrtorhinus lividipennis* adults on *Nilaparvata lugens* eggs.

Source of Variance	*C. lividipennis* Females			*C. lividipennis* Males		
	df	*F*	*p*	df	*F*	*p*
LTS	1	45.790	<0.001	1	2.874	0.098
Prey density	4	53.857	<0.001	4	15.525	<0.001
LTS × Prey density	4	2.454	0.061	4	1.260	0.302

**Table 2:** Maximum likelihood estimates (±SE) from logistic regressions of the proportion of preys consumed in relation to initial prey density by *Cyrtorhinus lividipennis* females and males exposed to LTS or not.

Sex	Treatment	Intercept	Linear	Quadratic	Cubic
Female	Control	1.7489 ± 0.5839 **	−0.0324 ± 0.0039 *	0.00361 ± 0.000738	−9.22 × 10^−7^ ± 2.21 × 10^−7^
	Post-storage	2.3849 ± 0.6302 *	−0.1648 ± 0.0009 *	0.00472 ± 0.000382 *	−4.23 × 10^−6^ ± 3.67 × 10^−7^
Male	Control	1.3847 ± 0.5972 **	−0.1911 ± 0.0017 **	0.00062 ± 0.000318	−8.23 × 10^−6^ ± 1.21 × 10^−6^
	Post-storage	5.3892 ± 0.4988 **	−0.2843 ± 0.0028 *	0.00156 ± 0.000213	−2.78 × 10^−6^ ± 5.41 × 10^−7^

* indicates a significant difference at *p* = 0.05; ** a significant difference at *p* = 0.01.

**Table 3:** Estimates of functional response parameters for *Cyrtorhinus lividipennis* adults predating on *Nilaparvata lugens* eggs.

Sex	Treatment	*R* ^2^	Attacking Rate (*α*) (/h)	Handling Time (*T_h_*) (h)
Female	Control	0.903	0.706 ± 0.06 b	0.020 ± 0.002 a
	Post-storage	0.870	1.006 ± 0.09 a	0.021 ± 0.002 a
Male	Control	0.905	0.751 ± 0.07 b	0.013 ± 0.002 b
	Post-storage	0.878	0.844 ± 0.08 a	0.021 ± 0.002 a

The data in the same column (mean ± SE) followed by different letters are significantly different between the control and post-storage adults of the same sex (*p* = 0.05).

## Data Availability

Data are available on request.
